# Yogi or fireball – or both – a diary study on the interaction between mindfulness and vigor on job performance

**DOI:** 10.3389/fpsyg.2024.1385674

**Published:** 2024-07-01

**Authors:** Johanna Barbara Blume, Jan Dettmers

**Affiliations:** Department of Work and Organizational Psychology, Faculty of Psychology, FernUniversität Hagen, Hagen, Germany

**Keywords:** dynamic performance, within-person analysis, vigor, mindfulness, human energy, personal resources, dairy study

## Abstract

**Introduction:**

Building upon the conservation of resources theory and the episodic process model of performance, this research addresses the gap in understanding how daily variations in two personal resources, particularly their interaction, affect job performance. Specifically, this study examines the influence of vigor and mindfulness on daily fluctuations in task performance considering the potential compensation effect between these personal resources in the workplace.

**Methods:**

We conducted a five-day online diary study involving 192 participants (926 daily observations). At the conclusion of each workday, participants were asked to assess their level of mindfulness and vigor in the workplace using validated scales, as well as estimate their task performance.

**Results:**

Multilevel analyses showed that both daily mindfulness and daily vigor positively predict self-reported task performance. The interaction between mindfulness and vigor was significant. The results suggest that high levels of mindfulness can compensate for low levels of vigor, and vice versa.

**Discussion:**

Exploring the interplay of personal resources at work provides a valuable starting point for individual-tailored interventions that enable individuals to reach their full potential. Enhancing employees’ mindfulness may increase job performance directly and empowers workers to compensate for periods of low energy.

## 1 Introduction

The significance of individual resources in the workplace is increasingly acknowledged. This is evident in the numerous and valuable contributions of theories such as the job-demands-resources theory and the conservation of resources theory to the analysis of the origins of stress, well-being, performance, and other critical outcomes in the workplace. Each employee possesses a unique set of personal resources that contribute to their daily functioning at work, enabling them to achieve their work-related goals (Halbesleben et al., 2014). According to the substitution hypothesis of conservation of resources theory, resources can serve as substitutes for each other when they align with environmental demands (Hobfoll et al., 1990). However, existing literature primarily focuses on examining the individual impact of specific resources on work outcomes.

To comprehensively understand the composition of an ideal set of personal resources, it is essential not only to explore their direct associations with work outcomes but also to investigate their dynamic interrelationships (Vermooten et al., 2021). The substitution hypothesis raises the question of which resources have the potential to compensate for each other. Existing research has identified external resources that can compensate for lacking personal resources in the workplace. For example, Ott et al. (2019) demonstrated that perceived organizational support (POS) could compensate for low levels of vigor and self-efficacy in relation to day-level work engagement. Similarly, Venz and Sonnentag (2015) and Venz et al. (2018) found that the strategy of selective optimization with compensation (SOC) can help employees manage a lack of role clarity and insufficient recovery.

However, these studies have not yet addressed whether personal resources can compensate for each other in an entirely internal and dynamic process. Given the increasing demands and stressors in modern work environments, understanding the compensatory effects of personal resources is crucial for enhancing employee well-being and performance. To address this research gap, our study aims to examine the compensatory effect of two well-established personal predictors of job performance, namely mindfulness and energy at work, at the daily level.

For that, we refer to the episodic process model by Beal et al. (2005), which incorporates a dynamic nature of performance. The model proposes that the allocation of resources – especially self-regulatory resources - is the primary mechanism that explains why employees perform well on 1 day and below their average the other day. Few attempts to test the proposition have identified resources such as positive and negative affective states (Merlo et al., 2018), and energetic state (Vahle-Hinz et al., 2021) as predictors of dynamic performance.

Unfortunately, personal energy resources deplete during work as well as during off-time (Dettmers et al., 2020) and are influenced by factors like sleep quality and recovery experiences (Hülsheger, 2016). To counteract the negative impact of low energy states on daily performance, we aim to investigate a potential antidote through the compensatory effect of another personal resource that can be enhanced when needed.

As the potential compensator, we focus on mindfulness – a state of attentiveness and awareness of present-moment experiences (Bishop et al., 2004; Brown et al., 2007). Mindfulness qualifies as a potential moderator, because it has been repeatedly claimed to improve self-regulation (Good et al., 2016). Additionally, it fluctuates naturally within individuals but can also be induced through brief daily mindfulness practices (Hülsheger et al., 2015).

In this study, we propose that the two personal resources – mindfulness and energy - can compensate for each other in regard to job performance because they are both associated with self-regulation and kick off similar cognitive, behavioral, and emotional processes. To test this assumption, we conducted a diary study investigating the joint effects of mindfulness and energetic activation on daily performance.

Our study makes several contributions to the literature. Firstly, we address the largely overlooked interaction between personal resources in the work context, specifically linking mindfulness and energy at work. This is important as it provides insights into the underlying processes through their interactive effect. Secondly, we contribute to understanding the short-term fluctuations of job performance within individuals. To our knowledge, this is the first study to examine state mindfulness and state energy at work as predictors of daily job performance within a common research model. This expands the field of empirical studies on the episodic process model proposed by Beal et al. (2005) and integrates research on mindfulness into the model. These insights are essential for a deeper understanding of how and when employees can reach their full potential at work. Furthermore, examining the interaction between personal resources at work can serve as a valuable starting point for developing tailored strategies to prevent and address issues related to low performance. For instance, it could provide justification for companies to support short mindfulness practices during work hours.

## 2 Theory and hypotheses development

### 2.1 Performance variability and vigor

Job performance is defined as employees’ behaviors at work that support organizational goals (Motowildo et al., 1997). In this article, we focus more specifically on task performance, which refers to “behaviors that are recognized by formal reward systems and are part of the requirements as described in job descriptions” (Williams and Anderson, 1991). Factors that predominantly account for differences in static job performance are relatively stable constructs like abilities, knowledge, experience, and non-cognitive traits like personality (Sonnentag et al., 2008). In the analysis of job performance, within-person variance has largely been treated as measurement error (Dalal et al., 2014). However, as postulated by the theory of dynamic performance (Beal et al., 2005), job performance is determined by stable and transient factors, such as day-specific job demands, resources and personal energy levels. Indeed, within-person fluctuations can explain approximately 50% of the variation in job performance (Podsakoff et al., 2019; McCormick et al., 2020).

In their model of dynamic performance, Beal et al. (2005) propose that resource allocation to the task and the direction of one’s attention to the task are central for momentary performance. This requires self-regulatory resources that are not stable but may fluctuate over time. Studies have argued that internal states comprising energy indicate that self-regulatory resources are available at a specific time, which leads to better performance (e.g., Vahle-Hinz et al., 2021). Energy is a sense of positive affective arousal, which includes an eagerness and capability to act (Quinn and Dutton, 2005). It is a concept involved in many motivational theories (Schippers and Hogenes, 2011), as research has shown that highly energized employees tend to be more motivated (Butt et al., 2020) and get work done – resulting in higher productivity (Chen et al., 2016).

To capture employees’ energetic resources, we focus on vigor as a much-studied concept, that captures the energy specifically directed at work and that may vary from episode to episode like job performance (Reis et al., 2016). Vigor is characterized by “high levels of energy and mental resilience while working, the willingness to invest effort in one’s work, and persistence even in the face of difficulties” (Schaufeli et al., 2006). It is one of three constitutive aspects of work engagement, reflecting a positive, fulfilling affective-motivational state of work-related well-being (Schaufeli et al., 2002).

Various studies have demonstrated the fundamental role of the current level of vigor for daily job performance. On a between-person level, Carmeli et al. (2009) have shown that vigorous employees tend to perform better according to supervisor ratings. At a within-person level, this relationship has not been studied decisively. However, related constructs indicating the energetic state have been found to predict task performance at a within-person level, for example, recovery state (Binnewies et al., 2009), fatigue (Dettmers et al., 2020), or the hourly measured energy level (Vahle-Hinz et al., 2021). We therefore propose Hypothesis 1:

*Hypothesis 1*: Daily vigor is positively related to daily self-reported task performance.

### 2.2 Mindfulness

Considering personal energy as a critical, limited resource that positively influences daily job performance and can become depleted (Parker et al., 2021), it is essential to explore the incremental impact of other internal states on job performance. Specifically, understanding the interaction between vigor and other internal states and how job performance is affected when vigor is low, is crucial. We focused on mindfulness as an internal state whose key feature is the self-regulation of attention (Good et al., 2016) and leads to higher functioning at work (Glomb et al., 2011). Therefore, we argue that it might be capable of buffering detrimental influences of low energetic states on momentary performance.

Mindfulness is a state of non-judgmental attentiveness and awareness of what is taking place in the present moment, internally and externally (Brown et al., 2007). One way to view mindfulness is as a personal resource that assists people in managing demands by directing their attention on the present moment rather than issues outside of their control (Grover et al., 2017). It has gained increasing attention in the context of work and organizational psychology (van Dam et al., 2018) as research on mindfulness has shown beneficial effects for employee health and performance (Lomas et al., 2017; Mesmer-Magnus et al., 2017). Workplace mindfulness has mainly been studied as a stable personality trait (e.g., Lyddy et al., 2021) or as a skill that can be trained through interventions (for a review, see Creswell, 2017). However, it has been repeatedly argued that mindfulness is at its core a state that naturally fluctuates from moment to moment within persons (state or daily mindfulness; Forjan et al., 2020).

Past studies have employed different operationalizations of mindfulness. However, most conceptualizations distinguish between two central components of mindfulness: awareness and acceptance (Bishop et al., 2004; Sauer et al., 2013). Nonetheless, Brown et al. (2007) argue that clear awareness of internal and external present-moment experiences is the first and foremost aspect of mindfulness. The authors define awareness as being aware of all physical senses as well as emotional and mental activities. In the context of organizational functioning, being aware seems especially valuable as it is a vital prerequisite for staying attentive and present while being engaged in a (work) task (Good et al., 2016). Accordingly, we want to follow most studies on mindfulness at work that measured mindfulness unidimensionally in the form of awareness – particularly when assessing state mindfulness in the workplace (Hülsheger et al., 2013, 2014; Haun et al., 2018).

In general, there is good evidence for mindfulness to play an essential role in performance. Mesmer-Magnus et al. (2017) reported meta-analytical evidence that employees high in trait mindfulness may work harder and perform better than employees low in mindfulness. However, understanding the state mindfulness’ role for organizational outcomes is insufficient (Forjan et al., 2020). Studies on naturally occurring short-term fluctuations of workplace mindfulness and how they relate directly to short-term performance variability are limited. To our knowledge, only Forjan et al. (2020) tested the intraindividual link at the day-level and found that on days when employees experienced heightened mindful awareness, they also reported stronger daily task performance. Similarly, Haun et al. (2020) reported a positive within-person correlation of awareness and goal attainment at work. We want to examine this relationship further and propose the following hypothesis based on these recent study results:

*Hypothesis 2*: Daily mindfulness is positively related to daily self-reported task performance.

### 2.3 Interaction of mindfulness and vigor on performance

In this study, we examine state mindfulness and state vigor as separate predictors of daily fluctuations in job performance. However, our main hypothesis suggests an interactive effect between these personal resources, beyond their individual impacts. Specifically, we propose the possibility of a negative interaction, whereby low energetic states can be compensated by high levels of mindfulness. This assumption is grounded in the shared underlying mechanisms through which both influence performance.

The positive effect of vigor on performance may be easily explainable by its energetic function: feeling vigorous, employees can approach their work tasks with more energy (Owens et al., 2016). Energy states indicate how much effort an employee is willing and ready to contribute (Schiuma et al., 2007). Energy is also involved in other motivational processes such as directing one’s effort to a work task and regulating the persistence in carrying it out (Owens et al., 2016). All in all, the capacity for action is increased in vigorous individuals so that work goals are achieved more easily (Owens et al., 2016). However, the linking mechanism is more complex: vigor is a positive experience *per se* as it is one aspect of positive affect (Shirom, 2011). Thus, notions about the association of positive affect with task performance are applicable.

In more detail, vigor may facilitate task performance through improved self-regulation and different mechanisms on cognitive, behavioral, and emotional levels: concerning self-regulation, some researchers consider energy as the accessibility of glucose in the blood, which determines the momentary capacity to self-regulate behavior (e.g., Gailliot et al., 2007). Following the model of Beal et al. (2005), self-regulation is the crucial factor for dynamic performance. Aside from self-regulation, Shirom (2003) argues that vigor generates a particular thought-action repertoire that expands activity and broadens the range of options. Furthermore, the broaden-and-build theory (Fredrickson, 2001) states that positive affects broaden the range of attention and cognition. Consequently, employees in a positive mood tend to see the big picture, take a holistic perspective, and show more approach tendencies (Diener et al., 2020). They also tend to have expanded cognitive repertoires, which leads to more problem-solving confidence (Forjan et al., 2020), efficient decision-making (Chuang, 2007), and increased creativity (Baas et al., 2008) – all potential facilitators of task performance.

Referring to mindfulness theories (e.g., Glomb et al., 2011; Good et al., 2016), we propose that mindfulness will trigger highly parallel mechanisms positively impacting job performance. Self-regulation of thoughts, emotions, and behavior is considered the central outcome of mindfulness with beneficial effects on performance (Glomb et al., 2011). This assumption is underlined by findings that electrophysiological features of mindfulness are associated with the self-regulation of attention (Verdonk et al., 2020) and the importance of self-regulation of attention to adequately concentrate on a task despite off-task concerns (Beal et al., 2005). Good et al. (2016) also emphasize improvements in stability, control, and efficiency of attention as the linkage between mindfulness and various other improvements in cognitive, emotional, and behavioral domains of functioning. Dane (2011) argues that broad attentional breadth held in the present moment is the key characteristic of mindfulness that influences task performance. In such a broad attentional state, employees are able to focus with due attention to the current task, but without losing their situational awareness (van Gordon et al., 2014). Additionally, mindfulness has been linked to cognitive capacity – especially in terms of improved working memory (Roeser et al., 2013) –, cognitive flexibility (Moore and Malinowski, 2009), and creativity (Baas et al., 2014). Glomb et al. (2011) argue that mindfulness fosters reduced automaticity of mental processes, which creates a mental gap between stimulus and behavioral response. This gives individuals response flexibility, which allows them to consciously choose behavioral responses to attain more adaptive outcomes (Good et al., 2016). In this way, the range and quality of possible reactions increases (Glomb et al., 2011). On a behavioral level, Weinstein et al. (2009) also found that mindful individuals confronted with stressful demands reported more approach than avoidance coping strategies. Furthermore, studies on mindfulness suggest that it leads to more positive and less negative affective reactions and quicker recovery from adverse work events (Glomb et al., 2011). It enables mindful people to avoid automatic, maladaptive emotional reactions and negative affective states by reflecting experiences from a meta-perspective (Malinowski and Lim, 2015). At this point, further beneficial effects of positive affective states may occur.

Considering all this, it becomes salient that mindfulness and vigor share many of the mechanisms in which they influence performance. First, both internal states influence job performance because they improve the capacity to self-regulate. The feeling of vigor indicates that self-regulatory resources are available, whereas mindfulness simplifies the act of self-regulation. This makes it easier for the employee to stay attuned to the current task. Second, vigor and mindfulness are accompanied by similar changes in cognition, behavior, and emotion (Good et al., 2016; Diener et al., 2020). Cognitively, both are associated with flexibility and breadth of mental processes. Behaviorally, both promote approach rather than avoidance strategies. Emotionally, both are either in themselves positive experiences or foster them.

According to the substitution hypothesis of conservation of resources theory (Hobfoll et al., 1990), personal resources are substitutable for each other. Both high state mindfulness and high state vigor can create a mindset that enhances employees’ performance. When this mindset is activated by one of these resources, improvements in performance may not arise from the other resource. However, if an employee is lacking either mindfulness or vigor, it is hypothesized that the other resource can compensate for the deficit and mitigate the decline in performance. Therefore, we assume a compensation effect:

*Hypothesis 3*: Day-level mindfulness and day-level vigor interact negatively on day-level self-reported task performance, in the form that one compensates for low levels of the other.

## 3 Materials and methods

To test our hypotheses, we conducted a diary study to capture the daily fluctuating states of vigor and mindfulness at work and related it to self-rated daily job performance. The diary study was conducted over five consecutive workdays. Each day, the participants had to complete short online questionnaires. The study variables were measured after work was finished. The participants received a link to the survey via e-mail according to the individualized schedule provided before the start of the study. After the five workdays, the participants completed a general survey that assessed sociodemographic data.

### 3.1 Sample

The criteria for study participation included regular employment with a minimum of 30 h per week. As this study was part of a major research project, another inclusion criterion was responsibility for at least one child aged 0–12 years living in the same household. This criterion was unrelated to our specific research question. We employed convenience sampling methods: Potential participants were contacted by social networks, such as XING and Facebook, and via personal contacts. To increase the motivation for participation, we promised to provide general feedback regarding the study results and an invitation to a workshop concerning the reconciliation of work and family duties. Furthermore, the participants received information about data protection regulations and the voluntariness of participation. They were asked to provide a formal informed consent to participate.

In total, 192 participants started the diary surveys resulting in 926 after-work surveys during the 5 days (34 after-work surveys missed). We further analyzed whether the actual measurement points corresponded with the predetermined survey schedule or not (e.g., a participant completing his after-work survey at night or next morning). In the latter case, we set the values of the respective study variables of that measurement point to “missing value.” In total, the dataset contained 848 after-work surveys of 189 participants completed at the correct time. In total, 52% of the participants were females. The mean age was 40 years (*SD* = 7.5), and 53% had a university degree. The mean working time indicated by the participants was 32 h per week (*SD* = 12). At the day-level, participants indicated on average 7.1 h.

### 3.2 Measures

In the daily after-work questionnaire, we assessed task performance, mindfulness state at work, and vigor. All items were assessed on a 5-point Likert scale ranging from 1 (*strongly disagree*) to 5 (*strongly agree*).

#### 3.2.1 Mindfulness

We measured the mindfulness state at the day-level with four items from the Mindful Attention and Awareness Scale [MAAS; Brown and Ryan, 2003; German translation by Michalak et al. (2008)]. A sample item is “I rushed through activities without being really attentive to them” (reverse scored). Reliability estimates of the daily measures based on Cronbach’s alpha ranged between *α* = 0.84 and *α* = 0.88.

#### 3.2.2 Vigor

We measured vigor with the three items vigor subscale of the UWES-9 (Schaufeli et al., 2006). A sample item is: “When I got up this morning, I felt like going to work.” Cronbach’s alpha ranged between *α* = 0.75 and *α* = 0.85 over the 5 days.

#### 3.2.3 Task performance

To assess daily task performance, we used four items from Williams and Anderson (1991; e.g., “Today, I adequately completed assigned duties”) to which the participants responded after work (t3). Cronbach’s alpha ranged between *α* = 0.89 and *α* = 0.92 over the 5 days.

#### 3.2.4 Control variables

We assessed daily work hours at the end of the workday as daily control variables because they potentially influence job performance (Ng and Feldman, 2008). Employees indicated how many hours they spent at work during the present day (*M* = 7.08; *SD* = 1.44). Table 1 displays the means and standard deviations of all study variables at the between and within-person level.

**Table 1:** Mean, standard deviations, and intercorrelations of the study variables.

		*M*_between_	*SD*_between_	*SD*_within_	1	2	3	4
	Day level							
1	Workhours	7.08	1.44	1.36	–	−0.08^*^	0.10^*^	0.00
2	Task performance	4.11	0.50	0.51	0.16^**^	–	0.40^**^	0.58^**^
3	Vigor	3.40	0.58	0.50	0.14^**^	0.41^**^	–	0.32^**^
4	Mindfulness	4.05	0.62	0.46	−0.03	0.40^**^	0.35^**^	–

Note: Within-person correlations are shown below and between-person correlations are shown above the diagonal. Number of observations: 848 with 189 participants. *p < 0.05; **p < 0.01.

### 3.3 Preliminary analysis

To examine whether the day-level measures of mindfulness, vigor and task performance were empirically distinct, we conducted Multilevel Confirmatory Factor Analysis (MCFA) via MPlus 8.4 (Muthén and Muthén, 1998–2017). The 3-factor day-level solution displayed acceptable fit to the data, *χ^2^* = 247.99, df = 84, *p* < 0.001, CFI = 0.95, TLI = 0.93, RMSEA = 0.04, SRMR = 0.04 (within level) and 0.13 (between level).

## 4 Results

We tested our hypotheses with hierarchical multilevel analysis using R. This study analyzed the relationships among the variables for which data were collected at multiple time points. We were primarily interested in within-person processes, i.e., the relationship in day-specific variation in vigor, mindfulness, and task performance. The share of within-person and between-person variation of the day-level variables derived from intercept-only models was investigated. The variance explained by the day-level (within-person variation) ranged from 46% (mindfulness) to 65% for task performance, indicating that a multilevel approach is justified. We centered the predictor variables vigor and mindfulness at the individual mean (person-mean centering).

To test our hypotheses, we compared several nested models. We tested the improvement of each model over the previous one by using the difference between the respective likelihoods. This difference follows a chi-square distribution, with the degrees of freedom corresponding to the number of parameters added to the model. Furthermore, all null models were tested for autocorrelation and heteroscedasticity. For all hypotheses, the null model included only the intercept controlling for effects of heteroscedasticity using the R package nlme (Pinheiro et al., 2021). For hypotheses H1, H2, H3, we computed a hierarchical regression model. Table 2 displays the results of the analysis.

**Table 2:** Results of multilevel analysis with task performance as outcome.

	Model 0			Model 1			Model 2			Model 3		
	Estimate	*SE*	*t*	Estimate	*SE*	*t*	Estimate	*SE*	*t*	Estimate	*SE*	*t*
Intercept	4.10	0.04	110.79^**^	4.10	0.04	110.73^**^	4.10	0.04	110.66^**^	4.11	0.04	111.51^**^
Workhours				0.06	0.02	3.90^**^	0.05	0.01	3.81^**^	0.05	0.01	3.79^**^
Vigor							0.29	0.04	7.97^**^	0.29	0.04	7.71^**^
Mindfulness							0.33	0.04	8.39^**^	0.31	0.04	7.53^**^
Vigor*Mindfulness										−0.14	0.06	−2.42^*^
−2*log likelihood		1692.79			1677.96			1501.89			1496.19	
Differences of −2*log					14.83			176.07			5.7	
Df		4.00			5.00			7.00			8.00	
*P*					0.00			0.00			0.02	

Note: *p < 0.05; **p < 0.01.

The results support hypothesis 1 and hypothesis 2 as vigor (*γ = 0.29, p* < 0.00) and mindfulness (*γ = 0.31, p* < 0.00) exhibited a significant within-person effect on task performance. Furthermore, the analysis revealed a significant negative interaction of vigor and mindfulness.

**Figure 1:**
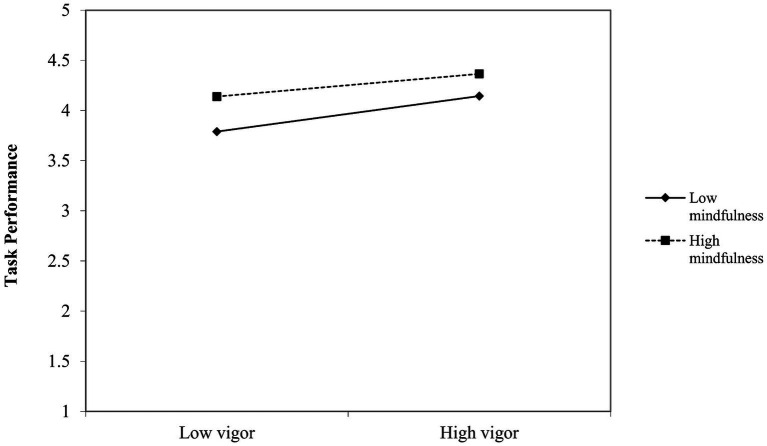
Simple slopes illustrating the vigor-mindfulness interaction on task performance.

**Figure 2:**
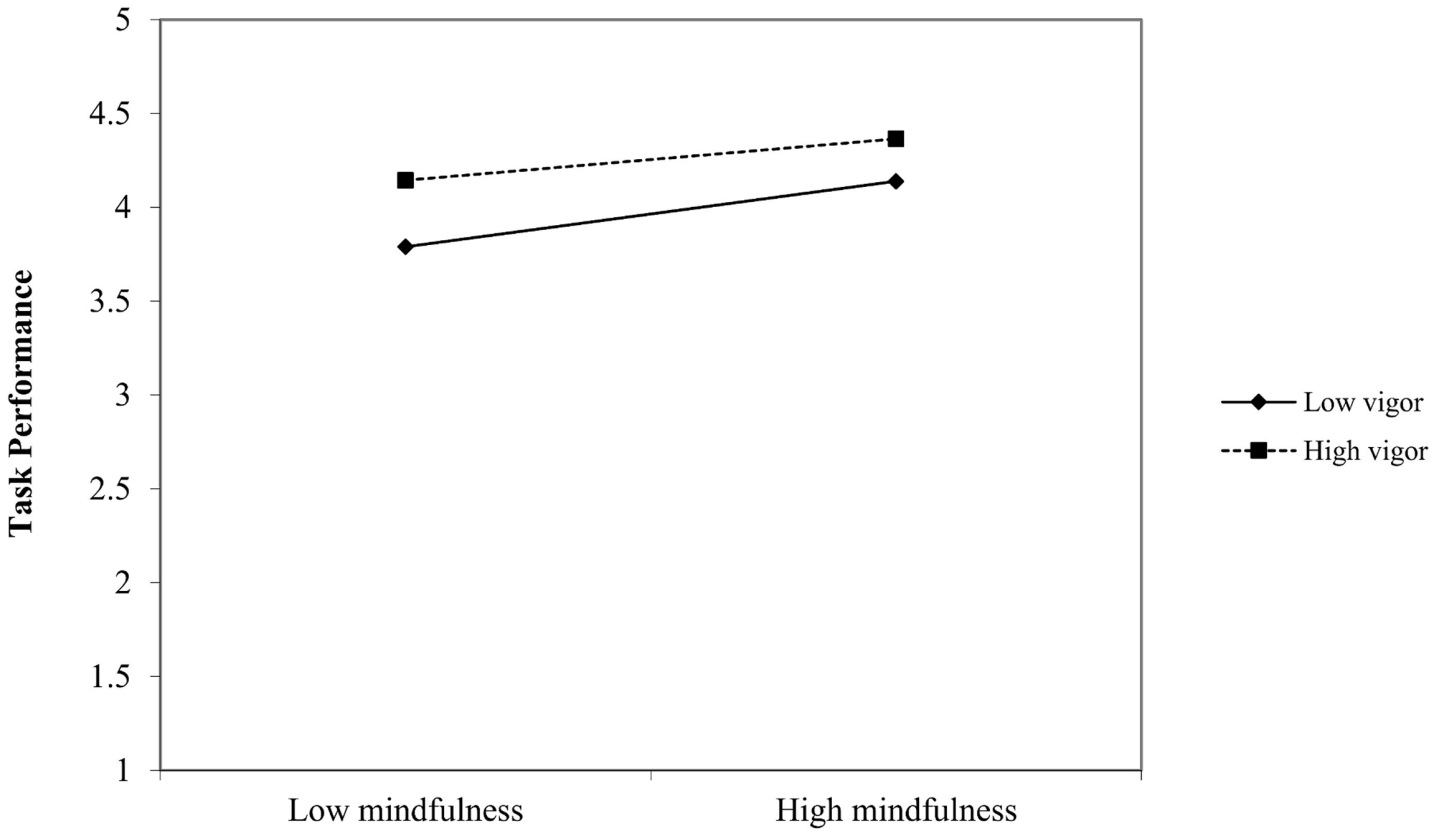
Simple slopes illustrating the mindfulness-vigor interaction on task performance.

For a more straightforward interpretation of the interaction effect, we conducted simple slope analyses. The results of the simple slope tests (Aiken and West, 1991) indicated that the slopes of vigor on task performance varied between days that participants experienced high mindfulness and days that participants experienced low mindfulness from 0.35 (unstandardized), *p* < 0.00 (days with low mindfulness) to 0.23 (unstandardized), *p* = 0.00 (days with high mindfulness). Conversely, the slopes of mindfulness on task performance varied between days where participants experienced high vigor and days where participants experienced low vigor from 0.38 (unstandardized), *p* < 0.00 (days with low vigor) to 0.24 (unstandardized), *p* = 0.00 (days with high vigor). Figures 1, 2 illustrate the differences in the simple slopes.

## 5 Discussion

This diary study sought to enhance our understanding of the influence of personal resources and their interplay in relation to fluctuating job performance. The present study supports that fluctuating levels of vigor and mindfulness are important internal predictors of daily performance at work. Consistent with hypothesis 1, the participants who reported feeling more vigorous rated their daily performance as better. That is particularly in line with Vahle-Hinz et al. (2021), who demonstrated that episodic energy levels were related to higher levels of self-reported episodic performance throughout one working day. Likewise, consistent with hypothesis 2, participants who experienced more mindfulness evaluated their day-specific performance higher. Thus, we replicate the results of Forjan et al. (2020), who also found a positive relationship of daily mindfulness and task performance. Those relationships had been argued theoretically and found empirically before on trait-level for vigor (Carmeli et al., 2009; Shirom, 2011) and mindfulness (Good et al., 2016; Lomas et al., 2017). However, we identified a shortage of empirical evidence regarding those relationships on the state level. This gap is unfortunate because the direction, shape, and size of a relationship on the state-level can differ from the same relationship on the trait-level (Dalal et al., 2014).

However, the core goal of this study was to test an interactive effect of vigor and mindfulness on task performance. Results suggest that the two personal resources interact, leading to a compensatory effect on performance. More precisely, on days when workers experience lower levels of vigor, the influence of mindfulness on task performance becomes more significant, while on days characterized by lower mindfulness, the impact of vigor on task performance gains greater importance. Put differently, when an employee experiences exhaustion and mindlessness on a particular day, their performance is likely to be negatively affected. Nevertheless, the presence of either vigor or mindfulness alone can compensate for the other, mitigating the current hindrance in state of mind. Consequently, this result reinforces our notion that the mechanisms of how vigor and mindfulness influence performance on a daily level may share important aspects on cognitive, affective and behavioral levels.

Theoretically, those findings add to the theory of dynamic performance (Beal et al., 2005) and its recent empirical tests by Merlo et al. (2018) and Vahle-Hinz et al. (2021). The presence of resources, particularly self-regulatory resources, determines an individual’s current work performance relative to their baseline level. Up to now the availability of self-regulatory resources in the sense of Beal’s model has been measured either by the valence component of affect (positive/negative affect; Merlo et al., 2018) or by its arousal component (energy; Vahle-Hinz et al., 2021). With mindfulness, we introduce a construct to the model that is not primarily an affective state - used as an indicator of self-regulatory resources – but one of which self-regulation is the central outcome (Glomb et al., 2011). This insight opens a new focus on variables to examine in the context of dynamic performance, as various constructs and conditions influence the current ability to self-regulate and therefore perform at one’s best.

Additionally, examining the potential compensatory effect of mindfulness on the relationship between low levels of vigor and task performance is a new research path. Only very limited research has explored same-level interactive effects, let alone compensatory effects, on dynamic performance. As an exception, Vahle-Hinz et al. (2021) found that task significance, as an environmental factor, can compensate for low energy levels in relation to task performance. Building upon this result, we contribute the insight that the detrimental effects of low energy on dynamic task performance can also be compensated for by another personal resource, namely mindfulness. Thus, this finding also supports the resource substitution hypothesis by Hobfoll et al. (1990).

### 5.1 Limitations and directions for future research

From a methodological point of view, our study has several limitations. First, all our variables were assessed with self-report measures and at the same measurement occasions (end of the working day). Therefore, common method bias may limit the validity of our results (Podsakoff et al., 2003). However, vigor and mindfulness are internal states and, as such, inherently subjective. Thus, they can hardly be assessed with other than self-report measures because of observability issues. To lower the concerns of common method bias that our findings are due to inter-individual traits, we centered the day-level predictor variables vigor and mindfulness at the individual mean (person-mean centering) so that all person-level variance was removed (Sonnentag and Niessen, 2008). Furthermore, the mean correlations of about 0.3 and the interaction effect give us confidence that our findings are not due to common method bias (Siemsen et al., 2010). For job performance, however, other measures exist (e.g., supervisor or co-worker ratings of performance, objective indicators), which are significantly less prone to social desirability in comparison to self-reports (Carpenter et al., 2014). However, in this study, we compare the daily performance rating of a person with their own performance ratings on other days so that self-presentational biases should be less of an issue (Beal et al., 2005). Moreover, employees usually know their daily tasks and objectives best in order to assess how far they have reached their goals, especially in such a short timeframe as 1 day (Kampf et al., 2021). Nevertheless, future research should use diverse measuring sources and separate and multiply measurement points throughout the working day (e.g., vigor and mindfulness in the morning and at noon).

Second, due to our correlational study design, causal conclusions cannot be drawn. Therefore, it remains unclear if vigor and mindfulness indeed increase job performance, as the relationship may also be reciprocal or reversed. For example, employees who observe significant achievements may experience increased vigor, while mindfulness may heighten awareness of minor tasks completed. Future studies could manipulate mindfulness (e.g., by mindfulness interventions before the workday) to investigate if the same results occur.

### 5.2 Practical implications

There is growing support for the notion that the comprehension of short-timeframe within-person performance variability has important implications regarding personnel development interventions (Dalal et al., 2020). In particular, it could help prevent short-term minimum performance called troughs (Dalal et al., 2014; Good et al., 2016). The results linking variability in both vigor and mindfulness to fluctuations in job performance offer potential avenues for addressing this issue. Both state mindfulness and state vigor independently impact performance, suggesting two approaches to enhance task performance. First, interventions could target the reduction of vigorless days. As day-level recovery is an important predictor of day-level work engagement (Sonnentag et al., 2010), recovery seems to be a fruitful approach for that. Therefore, job design should provide sufficient job resources to counterbalance job demands, reduce strain, and facilitate recovery (Demerouti et al., 2009). While this may raise mean levels of vigor and the frequency of invigorated days, there will always be less energetic days. In this case, mindfulness could function as a compensator. Again, there are possibilities to design jobs for mindful work (see Lawrie et al., 2018) and mindfulness trainings to raise trait mindfulness (Vonderlin et al., 2020). However, in light of our results, promoting the use of mindfulness tools when needed, such as mindfulness meditation practices (Hülsheger et al., 2013) can avert a working day marked by troughs. Just-in-time adaptive interventions could be a means for that (Nahum-Shani et al., 2018).

## Data availability statement

The datasets presented in this study can be found in online repositories. The names of the repository/repositories and accession number(s) can be found in the article. https://doi.org/10.23668/PSYCHARCHIVES.4974.

## Ethics statement

The studies involving humans were approved by Ethics Committee of the Medical School Hamburg. The studies were conducted in accordance with the local legislation and institutional requirements. The participants provided their written informed consent to participate in this study.

## Author contributions

JB: Writing – original draft, Formal analysis. JD: Writing – review & editing, Supervision, Project administration, Funding acquisition, Formal analysis, Conceptualization.
